# Improving access to gene therapy for rare diseases

**DOI:** 10.1242/dmm.050623

**Published:** 2024-04-19

**Authors:** Thomas A. Fox, Claire Booth

**Affiliations:** ^1^ UCL Institute of Immunity and Transplantation, University College London, London, NW3 2PP, United Kingdom; ^2^ Infection, Immunity and Inflammation Department, UCL Great Ormond Street Institute of Child Health, UCL, London WC1N 1EH, UK

**Keywords:** Gene therapy, Inborn errors of immunity, Inborn errors of metabolism, Rare disease

## Abstract

Effective gene therapy approaches have been developed for many rare diseases, including inborn errors of immunity and metabolism, haemoglobinopathies and inherited blindness. Despite successful pre-clinical and clinical results, these gene therapies are not widely available, primarily for non-medical reasons. Lack of commercial interest in therapies for ultra-rare diseases, costs of development and complex manufacturing processes required for advanced therapy medicinal products (ATMPs) are some of the main problems that are restricting access. The complexities and costs of navigating the regulatory environments in different jurisdictions for treatments that affect small numbers of patients is a problem unique to ATMPS for rare and ultra-rare diseases. In this Perspective, we outline some of the challenges and potential solutions that, we hope, will improve access to gene therapy for rare diseases.

## Teaser

**Summary:** Here, we summarise the issues surrounding access to gene therapy for rare diseases and explore potential solutions, and how they can be applied to improve access to these life-changing therapies.

## Introduction

**Fig. 1.**
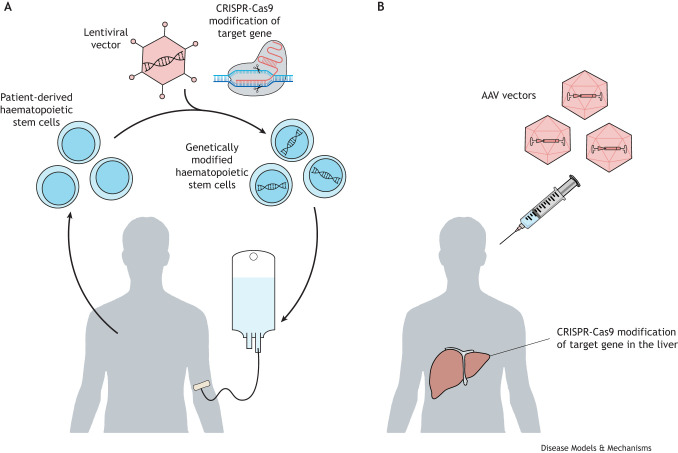
**Examples of *ex vivo* and *in vivo* gene therapies for rare diseases.** (A) *Ex vivo* gene therapy for rare diseases involves genetically modifying cells outside the body and then returning the cells back to the patient. For example, lentiviral vectors are used to transduce haematopoietic stem cells to correct adenosine deaminase-deficient severe combined immunodeficiency. (B) *In vivo* gene therapy involves genetically modifying cells inside the body by injecting a vector (e.g. virus or lipid nanoparticles); for example, adeno-associated virus (AAV) vectors are used for liver-targeted correction of haemophilia A.

The development of autologous gene therapy for inherited diseases is truly a remarkable success story. Although most genetic diseases are individually rare (defined as less than one in 2000 individuals), they are collectively common. It is estimated that one in 17 people are affected by a rare disease at some point in their lives (see: UK Government, Department of Health and Social Care, Policy Paper. The UK Rare Diseases Framework, 2021). Gene therapies are considered advanced therapy medicinal products (ATMPs), in which the DNA of a cell is engineered to restore or alter gene expression. Depending on the target tissue and technology being used, the genetic modification can occur either outside the body (*ex vivo*) with the cells then being returned to the patient or inside the body (*in vivo*) by injecting a vector (e.g. virus or lipid nanoparticles) (Fig. 1). For example, *ex vivo* haematopoietic stem cell gene therapy using lentivirus vectors has been developed to treat adenosine deaminase-deficient severe combined immune deficiency (ADA-SCID) and *in vivo* liver-targeted correction of haemophilia A has been developed using adeno-associated virus vectors (Kohn et al., 2021; Ozelo et al., 2022). This Perspective will focus on the issue of access to gene therapy but the current state of clinical development of gene therapy for rare diseases has been outlined in several recent comprehensive reviews (Mudde and Booth, 2023; Ferrari et al., 2023; Uddin et al., 2020).

Over the past five decades, proof-of-concept gene therapy approaches have been demonstrated for an array of inborn errors of immunity (IEIs) (Fox and Booth, 2020), metabolism (Gentner et al., 2021) and other inherited diseases, including haemoglobinopathies (Sagoo and Gaspar, 2023), cystic fibrosis (Allan et al., 2021) and congenital blindness (Hu et al., 2021) – with a few examples highlighted in Table 1.

**Table:**
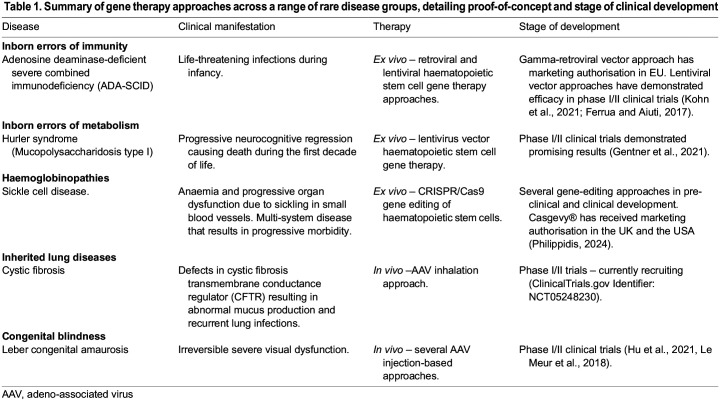
Table 1. Summary of gene therapy approaches across a range of rare disease groups, detailing proof-of-concept and stage of clinical development

Many of these approaches have progressed to clinical trials in humans and have demonstrated incredible efficacy that appears to result in long-term cures for devastating diseases (Gentner et al., 2021; Kohn et al., 2021; Gillmore et al., 2021). Several approaches have received marketing authorisation, including gene therapy products for haemoglobinopathies (approval of Casgevy® in 2023 in the UK and USA; see Philippidis, 2024), ADA-SCID (EU approval of Strimvelis® in 2016; see Hoggatt, 2016), retinal dystrophy (USA, EU, Canadian and Australian approval of Luxturna®; see Pennesi and Schlecther, 2020) and spinal muscular atrophy (USA, EU, Canadian and Australian approval of Zolgensma®; see Bitetti et al., 2023). Despite this remarkable progress, gene therapy for rare diseases is not widely available, even in advanced healthcare systems, due to non-medical reasons (Aiuti et al., 2022; Fox et al., 2023). The reasons for this are complex but are primarily related to the high costs of development and manufacturing of the therapies, alongside regulatory and marketing authorisation challenges, and small market population.

## Barriers to accessing gene therapy

Drug development is an expensive endeavour. It has been estimated that the total cost of developing a new ATMP is up to $1 billion. Such stratospheric figures have limited drug development from academic developers to commercial pharmaceutical companies, who recoup these costs through the sale of drugs to large market populations (Vernon et al., 2010). These costs do not include external pre-clinical research in academia on disease mechanisms or the therapeutic approach itself (Avorn, 2015). Over half of the cost of drug development is dedicated to funding the clinical development of the drug – from clinical trials to marketing approval (Avorn, 2015; Vernon et al., 2010). However, despite the high costs of ATMP development, some studies suggest that gene and cell therapies are being priced to deliver profit margins far greater than those needed to recoup the costs of research and development. For example, a recent study estimated that, in the USA, the current pricing of Tisagenlecleucel (Kymriah®) – a chimeric antigen receptor T cell therapy for haematological malignancies – generates an 84% profit margin over 10 years for Novartis (Kleutghen et al., 2018). Increased application of value-based pricing mechanisms could help address this issue; it is notable that the costs of ATMPs are higher in the USA where – compared with other countries – there is no regulation of medicine prices at market launch (Jommi et al., 2020; Goncalves, 2022).

The current model for drug development has two main implications for developing gene therapies for rare diseases. First, even for approaches fully developed up to the clinical trial stage in academia, a large amount of capital is needed to fund the clinical development process and obtain marketing authorisation. This ordinarily necessitates the involvement of a commercial partner. Second, given that pharmaceutical companies are run with a for-profit business model, investment decisions are based on the likelihood of a given product generating a return on investment. Therefore, developing drugs for many rare diseases is simply not an attractive or feasible commercial proposition. We have recently observed widespread disinvestment in the field of IEIs. These life-threatening inherited diseases of the immune system are classed as ultra-rare diseases (affecting fewer than one in 50,000 people). Gene therapy approaches for some IEIs have reached advanced stages of clinical development with excellent long-term safety and efficacy data (Reinhardt et al., 2021). Despite this, Orchard Therapeutics, the commercial partner for the first IEI gene therapy product to receive marketing authorisation (Strimvelis®), recently withdrew from that programme (Fox et al., 2023; see also: Orchard Therapeutics Extends Runway into 2024, Focusing HSC Gene Therapy Platform Exclusively on Severe Neurometabolic Diseases and Research Platform). A further blow to the availability of gene therapies for rare diseases occurred in 2021 when bluebird bio, a company that makes gene therapies for rare diseases, including cerebral adrenoleukodystrophy and beta thalassaemia, completely withdrew from the European market, citing difficulties in agreeing on reimbursement with European authorities as the reason behind the withdrawal (see: Bluebird, winding down in Europe, withdraws another rare disease gene therapy). Authorities in Germany, for example, refused to cover the $1.8 million price tag for gene therapy by using Zyntelgo® (bluebird bio) to treat beta thalassaemia, highlighting the challenges in bringing gene therapies for rare diseases to market (see: Bluebird, winding down in Europe, withdraws another rare disease gene therapy).

In the rare disease setting, small market populations – and competition from existing more-toxic treatment approaches, such as allogeneic haematopoietic stem cell transplantation – make expensive gene therapy approaches an unattractive commercial proposition

In the rare disease setting, small market populations – and competition from existing more-toxic treatment approaches, such as allogeneic haematopoietic stem cell transplantation – make expensive gene therapy approaches an unattractive commercial proposition. The for-profit approach in this field has become a source of much frustration for patients and physicians. Despite excellent safety and efficacy data, patients are unable to access and benefit from safer autologous gene therapy approaches for non-medical reasons.

For several rare diseases, commercial companies are still taking gene therapy products towards marketing authorisation. Examples include neurometabolic disorders (such as metachromatic leukodystrophy, mucopolysaccharidosis types I and IIIA) and haemoglobinopathies. These diseases are either more common, such as sickle cell anaemia, or have no effective alternative treatment options, making gene therapy a more commercially viable prospect, as is the case for spinal muscular atrophy. However, despite commercial interest, the current model is still failing patients and healthcare systems.

To return a profit in the rare disease setting, the cost of therapy is, at first glance, astronomically high. Zolgensma®, the gene therapy for spinal muscular atrophy, made headlines as the world's most expensive drug when it received approval in 2019 (Nuijten, 2022), with a list price of £1.79 million per treatment (see NHS England: NHS England strikes deal on life-saving gene-therapy drug that can help babies with rare genetic disease move and walk, 2021). A new gene-edited therapeutic approach for sickle cell anaemia and thalassaemia, i.e. exagamglogene autotemcel (Exa-Cel), known as Casgevy® in Europe, which recently received marketing authorisation in the UK and USA is priced at $2.2 million – for the drug alone – in the USA (The Lancet, 2023). The costs of these gene therapies will limit availability to the most resource-rich healthcare settings. Even in high-income countries, such therapies will place a strain on healthcare systems and rationing of therapy will be needed.

Indeed, even in the context of more common disease settings, such as cancer, there is concern that the high costs of ATMPs is affecting access to drugs. Chimeric antigen receptor T (CAR-T) cells have produced dramatic results in common haematological malignancies, such as diffuse large B cell lymphoma and acute lymphoblastic leukaemia (Maude et al., 2015; Maude et al., 2018). There is widespread commercial interest in CAR-T cell therapies and five different CAR-T cell products have received marketing authorisation in the USA, with many more expected to follow (see: U.S. Food and Drug Administration, Approved Cellular and Gene Therapy Products). Despite the large market, CAR-T cell therapy remains expensive, costing over $350,000 per product per patient (Fiorenza et al., 2020). Cost effectiveness analyses for treatments commonly use quality adjusted life years (QALYs) to provide a composite measurement of morbidity and mortality (Porter et al., 2015; Porter, 2010), with a year of life lived in perfect health being worth one QALY. The national health insurance programme in the USA, Medicare, has a willingness-to-pay threshold of between $100,000 and $150,000 per QALY. Depending on the indication and, thus, response rate, CAR-T cell therapy costs between £100,000 and £170,000 per QALY, suggesting that the cost of treatment is at the upper limit of what advanced health care systems are able to bear (Fiorenza et al., 2020).

Gene therapies for rare diseases are unlikely to meet affordability thresholds given their multi-million-dollar price tags. There is a strong argument in favour of one-time curative gene therapies that have a higher cost-effectiveness threshold, and the Institute for Clinical and Economic Review (ICER) in the USA has recently discussed a threshold of $500,000 per QALY for rare and ultra-rare diseases (Garrison et al., 2019). However, for most new gene therapies, the duration of action is yet to be determined, thus placing healthcare providers in a difficult position when assessing the cost effectiveness of a new, expensive therapy. However, a one-time gene therapy may be cost effective, when compared to a lifetime of expensive non-curative treatment, for instance enzyme replacement for patients with ADA-SCID or factor replacement for patients with haemophilia, alongside the costs of managing progressive morbidity associated with chronic disease. To address the uncertainty faced by health technology-assessment bodies and -reimbursement agencies regarding short-term clinical data for gene therapies, new innovative payment mechanisms have been proposed (Jorgensen and Kefalas, 2021). Payments related to short- and long-term outcomes, so called ‘payment by results’, is one potential solution to the uncertainty around long-term efficacy and large up-front costs of gene therapy approaches (Jorgensen and Kefalas, 2021).

## Solutions

### Reducing the manufacturing cost of gene therapies

Even when the development costs are removed, manufacturing ATMPs is expensive (Harrison et al., 2019). The bespoke nature of most cell and gene therapy means that economies of scale cannot be realised. The manufacturing process on a by-patient basis means that many critical steps, such as analytical testing, quality control and assurance, depend on skilled human labour (Riviere and Roy, 2017). As the manufacturing infrastructure for cell and gene therapies expands, increased competition will drive reductions in facility costs (Harrison et al., 2019). Technological advances are enabling increased automation of the manufacturing process and further innovations in this area will reduce the amount of hands-on skilled human labour needed to manufacture each product (Ramanayake et al., 2015). Furthermore, advances in cryopreservation have expanded the limited geography of production and delivery of cell and gene therapy (Kohn et al., 2021; Arlabosse et al., 2023). Offshoring manufacturing to locations with lower staff and facility costs is one way in which production costs could be further reduced (Harrison et al., 2019). However, efforts would need to be made to ensure that such offshoring does not just take advantage of a lower-paid workforce but also benefits the countries in which the manufacturing is taking place. Such activities potentially have the additional benefit of increasing accessibility and infrastructure for delivery of ATMPs in these markets. Owing to these and other developments, it is anticipated that production costs for gene therapies will fall soon. However, although cost-savings can and will be made in the manufacturing process, the cost of viral vectors for gene therapy are likely to remain prohibitively expensive for the foreseeable future. The cost of vector manufacturing alone for an adeno associated viral vector (AAV) for example, is $1–2 million USD per dose (see rolandberger.com/en/Insights/Publications/Cutting-the-cost-of-gene-therapy-manufacturing.html). Unfortunately, the personalised nature of each therapy means that costs for ATMPs will always be higher than off-the-shelf medicines.

### Streamlining regulatory approvals

Current regulatory evaluation systems are designed around mass produced small molecules and traditional drug compounds, and do not consider the unique properties of ATMPs for rare diseases (Aguilera-Cobos et al., 2022). There is often also a lack of recognition of approvals between different jurisdictions and differences in assessment criteria between jurisdictions, which makes seeking approvals challenging and expensive. This is particularly the case when pursuing approval through chemistry, manufacturing and controls management due to the need to navigate complex regulatory landscapes and country-specific stipulations (see: Understanding regulatory submission and the role of regulatory CMC project management). Exceptions have been made for *n*=1 diseases, allowing a fast-track route to approval on a case by case basis; moreover, it could be argued that similar bespoke criteria should be applied to ultra-rare diseases affecting, at most, a few hundred patients worldwide (Aiuti et al., 2022; Kim et al., 2019). In the case of a patient-specific oligonucleotide therapy, the therapy was approved by the U.S. Food and Drug Administration (FDA) under the new drug application (NDA) pathway. Previously, the FDA approved the repurposing of existing drugs for seriously ill patients without other treatment options, but this was the first time this pathway had been used to approve a new patient-specific therapy (Kim et al., 2019). Platform approvals are another way in which the regulatory approval process could be streamlined. Platform approvals refer to the approval of a gene therapy vector, with the approvals permit allowing the gene of interest to be changed. Given that many gene therapies for different diseases are built around a near-identical ‘platform’ vector, recognition of these similarities could de-risk and accelerate the development of bespoke gene therapies for rare diseases (see AgencyIQ – CBER's Peter Marks on advancing gene therapy, using AI, pushing accelerated approval and bespoke platforms).

The recognition of quality assessment between regulatory agencies and bespoke criteria are some of the ways in which the regulatory burden could be eased (Aiuti et al., 2022; Fox et al., 2023). As regulatory agencies are aware of the unique difficulties faced by ATMPs for rare diseases, they are taking action to try to mitigate current barriers. The European Medicines Agency, for example, has recently launched a pilot program to offer enhanced support to academics and non-profit developers of ATMPs (see ejprarediseases.org/ema-pilot-offers-enhanced-support-to-academic-and-non-profit-developers-of-advanced-therapy-medicinal-products/2022). This pilot program will offer enhanced regulatory support for non-profit academic developers of ATMPs by addressing unmet clinical needs. A further advance in this area is that regulatory agencies are more open to referring to expertise and decision making of trusted regulatory authorities in other jurisdictions. For example, in 2023 the UKs Medicines and Healthcare Products Regulatory Agency (MHRA) announced that, from 2024, they would recognise foreign approvals from trusted authorities − in this case the regulatory authorities of Australia, Canada, the European Union, Japan, Switzerland, Singapore and the USA – in their own decision making (see MHRA announces new recognition routes to facilitate safe access to new medicines with seven international partners).

### Innovative payment mechanisms

Novel reimbursement models are a potential solution to the high up-front costs of curative gene therapy and the uncertainty surrounding the duration of response for a new ATMP (Jorgensen and Kefalas, 2021). Health technology assessment (HTA) bodies are increasingly presented with ATMPs that theoretically have lifelong efficacy but only comprise clinical trial data with several years follow up (Goodman et al., 2022). This has driven the formation of outcome-based reimbursement (OBR) schemes. Most of the OBR schemes to date have been used in the context of cell therapies for cancer, e.g. chimeric antigen receptor T cells, which differ from potentially more expensive curative gene therapies for rare diseases (Jorgensen and Kefalas, 2021). However, the principles of these schemes could be used in the context of rare diseases.

There are different OBR schemes in European countries. All involve the collection of real-world data to enable further assessment of efficacy during the period following marketing approval (Jorgensen and Kefalas, 2021). OBR schemes, such as reimbursement agreement for *Zolgensma*® in Germany, can include up to 100% reimbursement of the drug cost based on patient-relevant outcomes (see: Vertragsabschluss zwischen AveXis und der GWQ zur erfolgsorientierten Erstattung von Zolgensma®). Other schemes, such as the reimbursement arrangement for *Yescarta*® in Italy, include payment in instalments subject to sustained outcomes – in this case remission of B cell lymphoma (https://www.navlindaily.com/article/2748/gilead-s-yescarta-reimbursed-in-italy-via-three-installment-payment-at-results-model). Whilst innovative new payment systems can help reimbursement arrangements for expensive gene therapies, these systems need to be accompanied by regulatory reforms that permit departure from volume-based contracting for drugs (Daniel et al., 2017). OBR schemes raise issues related to patient privacy, i.e. data on outcomes need to be shared with authorities, and government price reporting, i.e. companies report based on per-unit prices. These potential regulatory hurdles need to be addressed as OBR schemes are conceived, to allow manufacturers of gene therapies to remain compliant with regulations (Daniel et al., 2017).

### Hospital exemption pathway

**Fig. 2.**
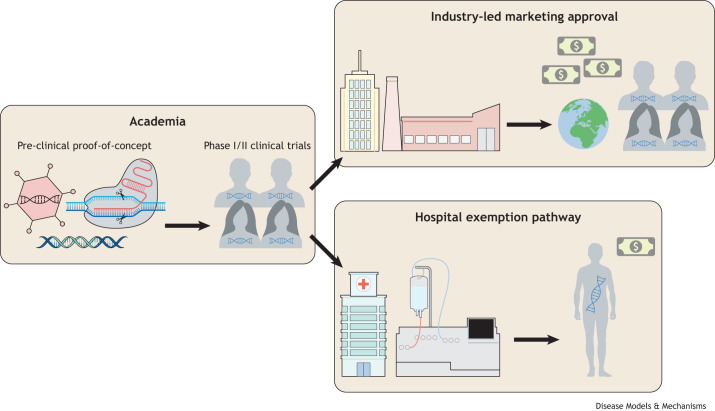
**Different pathways to deliver a gene therapy outside of a clinical trial.** Many gene therapies for rare disease are developed in academia, subsidised by public and charitable funding. Pre-clinical proof-of-concept and academic clinical trials generate a comprehensive data package. This can catalyse industry-led market approval or inform the hospital exemption pathway. In the industry setting, the company then sells the product for a profit to recoup development costs. In the hospital exemption pathway, the product can be administered on a per-patient basis. In this scenario, academic medical centres have responsibility for quality standards, traceability and pharmacovigilance standards.

The ‘hospital exemption’ (HE) pathway is the only alternative to the market authorisation pathway for providing new therapeutic products to patients outside of clinical trials in the EU (Fig. 2) (Ott de Bruin et al., 2023; Trias et al., 2022). The HE pathway enables an ATMP to be prepared on a non-routine basis for an individual patient and to be used only within the state in which it was manufactured. Quality standards, traceability and pharmacovigilance standards need to be met and the administering hospital site retains responsibility for this (Trias et al., 2022). Proof-of-principle of this approach has recently been demonstrated by using chimeric antigen receptor (CAR) T-cells (Castella et al., 2018). The CD19-directed CAR-T cell product ARI-00 established by academic developers received approval under the HE pathway for treating patients with acute lymphoblastic leukaemia and was re-imbursed by the government (Trias et al., 2022; Ortiz-Maldonado et al., 2021; Juan et al., 2021). A similar approach could be used for gene therapy for rare diseases. The provision of gene therapies by academic medical centres on individual patient basis, would significantly reduce the financial burden as the products would be produced at-cost rather than for-profit.

In the UK, unlicensed medical products, such as ATMPs, can be manufactured or imported under the MHRAs ‘specials’ license to treat an individual patient. This procedure has strict criteria that need to be met and products need to be supplied on a named-patient basis. Whilst such a route is inappropriate for medicines required at higher scale, within the ultra-rare disease setting, this regulation could be used for effective therapies that have not yet progressed through licensing (see https://www.gov.uk/government/publications/supply-unlicensed-medicinal-products-specials). A related new model has recently been established in Italy for *Strimvelis*®. Following the disinvestment in IEIs by Orchard Therapeutics, the Italian research charity Telethon took over the license to produce and distribute *Strimvelis*® (Valsecchi, 2023).

The access to gene therapies for rare diseases (AGORA) foundation was recently founded by clinicians, academics and a patient advocacy group with the aim to find solutions to the challenges of accessing effective gene therapy for rare diseases […].

### AGORA initiative

The Access to Gene Therapies for Rare Diseases (AGORA) foundation was recently founded by clinicians, academics and a patient advocacy group with the aim to find solutions to the challenges of accessing effective gene therapy for rare diseases outlined in this article. This foundation now also engages with diverse stakeholders, including other patient organisations and industry representatives (Fox et al., 2023). AGORA aims to act as a central facilitator across Europe and the UK to support national regulatory submissions, Health Technology Assessment and infrastructure readiness, as well as a network of expert academic medical centres to aid the delivery of proven gene therapies. The focus of the initiative is cross border collaboration and data sharing to develop a pipeline approach that enables patient access to therapies, agnostic of platform.

## Conclusions

At the current time, patients with rare and ultra-rare diseases are unable to access safe, proven and effective gene therapies. Without significant changes in drug development and authorisation pathways this situation will continue to frustrate patients, their families and clinicians alike. Fortunately, stakeholders are engaged in bringing about changes to improve access to gene therapy. We are optimistic that this work will enable more patients with rare diseases to benefit from these transformative gene therapies.
